# Effect of dietary supplementation of *Lawsonia inermis* and *Acacia nilotica* extract on growth performance, intestinal histopathology, and antioxidant status of broiler chickens challenged with coccidiosis

**DOI:** 10.1186/s12917-024-04409-w

**Published:** 2025-01-06

**Authors:** Fares A. Eldeeb, Enas A. Noseer, Shimaa Abdelazeem, Esraa Ali, Asmaa W. Basher, M. A. A. Abdalla, Hytham H. Ibrahim

**Affiliations:** 1 Department of Nutrition and Clinical Nutrition, Faculty of Veterinary Medicine, Aswan University, Aswan, 81528 Egypt; 2 Department of Biochemistry, Faculty of Veterinary Medicine, Aswan University, Aswan, 81528 Egypt; 3 Department of Nutrition and Clinical Nutrition, Faculty of Veterinary Medicine, South Valley University, Qena, 83523 Egypt; 4 Department of parasitology, Animal Health Research Institute (AHRI), Agricultural Research Centre (ARC), Qena branch, Qena, Egypt; 5 Department of pharmacology, Faculty of Veterinary Medicine, South valley University, Qena, Egypt; 6 Department of Pathology, Faculty of Veterinary Medicine, Zagazig University, Zagazig, Egypt; 7 Department of Poultry and Rabbit Diseases, Faculty of Veterinary Medicine, Aswan University, Aswan, 81528 Egypt

**Keywords:** *Acacia nilotica*, Antioxidants, Coccidiosis, Cytokine, Growth performance, *Lawsonia inermis*

## Abstract

Avian coccidiosis is one of the many disorders that seriously harm birds’ digestive systems. Nowadays the light is shed on using Phytochemical/herbal medicines as alternative natural anti-coccidial chemical-free standards. Consequently, this study aimed to investigate the impact of *lawsonia inermis powder* (*LIP*), and *Acacia nilotica* aqueous extract (*ANAE*), on growth performance, serum biochemical, antioxidant status, cytokine biomarkers, total oocyst count and intestinal histopathology of broiler chickens challenged with coccidiosis. Two hundred and forty-one-day-old Ross chicks were randomly distributed into 8 groups, four were challenged with coccidia, while the other four were unchallenged. Each group consisted of 3 replicates of 10 chicks each. The birds were challenged with *Eimeria species* orally on day 14 of age. Group 1B was unchallenged, and Group 2 A was challenged with coccidiosis and both were fed the basic diet without additives. Groups 3 A, 4 A, and 5 A were challenged and fed on the basic diet supplemented with *LIP* (40 g/kg of diet), *ANAE* (5 g/kg of diet), and *LIP* + *ANAE* combination, respectively. Groups 3B, 4B, and 5B were unchallenged and fed on the basic diet supplemented with *LIP* (40 g/kg of diet) and *ANAE* (5 g/kg of diet) and *LIP* + *ANAE* combination, respectively. The best results of growth performance parameters were recorded in G5B, and G5A followed by the group fed on *ANAE* and then the group fed on *LIP* compared with the control. All challenged broilers had higher aspartate aminotransferase (AST), alanine transaminase (ALT), urea, creatinine, glucose, MDA, IL-4 &TNF-α levels compared to all unchallenged broilers. Challenged broilers had lower serum cholesterol, triglycerides, total protein, albumin, globulin, SOD, GPX & IL-10 levels compared to non-challenged broilers. Histopathological examination of the small intestine and cecum of challenged treated groups with *LIP* + *ANAE* showed good mucosal integrity, few leukocytes infiltration, and low total oocyst count in broilers manure, followed by *ANAE* then *LIP* groups. In conclusion, dietary supplementation of *lawsonia inermis powder* and *Acacia nilotica* aqueous extract either alone or in combination had positive effects on broiler performance, blood metabolites, antioxidant status, cellular response, and intestinal morphology during the exposure to *Eimeria* spp. as a potential natural anti-coccidial.

## Introduction

 It is imperative to find alternative feed additives that can enhance gut health of poultry, reduce the severity of disease and be more affordable than antibiotic growth promoters (AGPs), as AGPs are a common ingredient in commercial poultry diets and are becoming more and more harmful to both human and avian health. Avian coccidiosis is caused by several species of the Eimeria protozoan, which costs the poultry industry US$14 billion a year in treatment, lost production, and administrative expenses [1].

The main four principal coccidiosis species, *E. tenella*,* E. necatrix*,* E. acervulina*,* and E. maxima*, are causing substantial financial losses in the poultry industry. Anorexia, decreased nutritional digestibility, reduced efficiency of nutrient utilization, and expenditure of nutrients to repair injured tissues are all caused by coccidiosis, which also results in decreased avian growth performance [2]. Additionally, coccidia can cause oxidative stress, leading to the destruction of cell membranes, increased their permeability, and reduced their functionality. These damages cause a chain of events that have a lasting effect on the health of the chickens or perhaps cause high morbidity & mortality [3]. Super oxide dismutase (SOD) and glutathione peroxidase (GPX) are two antioxidant enzymes that are vital for animal defense mechanism against oxidative damage [4].

Nowadays, medications and vaccinations are still used in the prevention and treatment of coccidiosis. Vaccines have a limited ability to suppress infection, and the use of anti-coccidial medications can result in the establishment of drug-resistant strains [5]. Moreover, some nutrients and energy were needed to create immune response cells, which diverted them from the anabolic process and altered metabolism, in order to activate and maintain the immune system [6].

Two distinct cells populations that produce interleukins (IL-4 and IL-10) are responsible for two different types of immune responses in poultry infected with coccidiosis to improve the parasite’s control via antibody-mediated cytotoxicity [7].

Herbal products have received global attention in recent times for their potential to manage multiple diseases while lowering the development of resistance [8]. More than 1200 plants have been found to have antiprotozoal properties. Some of these herbal remedies are employed in poultry diets because of their natural ability to stimulate the immune system and promote growth [1]. Fortunately, natural herbal extracts have many pharmacological effects, including anti-inflammatory and antibacterial properties that may help in treatment of coccidiosis, and they also have great antioxidant capabilities [9].

Henna is a subtropical and tropical plant with the scientific name *Lawsonia inermis*. Mucilage, mannite, gallic acid, and tannic acid are the primary chemical components of henna [10]. Henna is an immune stimulant [11], anti-inflammatory, tuberculostatic [12], antimicrobial, anti-parasitic anticancer, with antioxidant properties [10] used in traditional medicine to treat a wide range of diseases including hepatitis, leprosy, pain, spleen enlargement, dysentery, and dermatological conditions [13].

In tropical and subtropical areas, there is a medicinal plant called *Acacia nilotica*. The bioactive chemicals in *Acacia nilotica* are abundant, and it is used to prevent and treat a wide range of illnesses including infectious disorders. The plant is abundant in phytochemicals such as tannins, saponins, glycosides, and flavonoids, which are substances capable of producing a variety of physiochemical and pharmacological effects and managing a variety of disorders [14]. Due to the presence of multiple phytochemicals with diverse antioxidant, cytoprotective, and anti-inflammatory effects, *A. nilotica* seed was linked to edible and nutritious qualities [15].

The goal of the current study was to investigate the effect of dietary supplementation of *Lawsonia inermis***(**Henna**)** powder, and an aqueous extract of *Acacia nilotica* pods either alone or in combination on growth performance parameters, serum biochemical metrics, antioxidant status, cytokine biomarkers, total oocyst count and intestinal histopathology of broiler chickens challenged with coccidiosis.

## Materials and methods

### Feed additives

Commercial *Lawsonia inermis* (Henna) powder and *Acacia nilotica* pods were obtained from herbalist stores in Aswan, Egypt. *Lawsonia inermis* powder (LIP), was added to broiler diets at starter, grower and finisher phase starter of broilers’ diet at a dose rate of 40 g/kg without any deleterious effect on growth performance, hematology and serum biochemical parameters as recommended by Adedeji et al. [16].

### Preparation of *Acacia nilotica* aqueous extract (ANAE)

*Acacia nilotica* aqueous extract (ANAE) was prepared in accordance with the procedure outlined by Raheel et al. [17]. The pods were ground into fine powder using electric mixture and subsequently soaked in double distilled water (DDW) at a ratio of 1:4 at 4 °C for 5 days. The resulted extract was filtered using autoclaved filter papers (Whatmann No. 1, 0.45 μm pore size) [17]. ANAE was added to the broilers’ diet at a dose of 5 g/kg as recommended by Zahid et al. [18]. The feed additives were mixed with a small amount of diet and then mixed this with the main bulk of diet to achieve thoroughly mixing and dose formulation.

### Preparation of coccidial inoculants

Fresh fecal samples of *coccidiosis* infected broilers were collected to isolate the *Eimeria* species sporulated oocysts in Parasitology Lab of the Animal Health Research Institute (AHRI), Qena, Egypt using the method described by Chand et al. [19]. The fecal samples were mixed with saline solution in 2-ml microfuge tubes. The supernatant was discarded after centrifugation of the samples for 5 min. The oocysts were allowed to sporulate in 2.5% (w/v) aqueous potassium dichromate solution (K_2_Cr_2_O_7_) for a week at room temperature (23–25 °C). Throughout this duration, the oocysts were inspected several times, and the sporulation time was noted [20].

### Coccidiosis challenge model

An infectious dose of 30,000 Eimeria species sporulated oocysts per 2 ml of inoculum was used. At 14 days old, each bird of the challenged groups received 2 ml of the parasite suspension separately using a crop needle according to [17, 21]. However, unchallenged birds were given 1 ml of sterile normal saline instead of the parasite suspension.

### Experimental birds and design

Two hundred and forty-one-day-old, unsexed chicks (Ross 308) were purchased from Al Wadi Poultry Company, ‏‏‎Sadat City, Monufia Governorate, Egypt with an average initial body weight of 43.05±0.91 g. The birds were weighed individually and then assigned randomly to 8 treated groups. Four of these groups were challenged with coccidia, while the other four groups were unchallenged. Each group consisted of 3 replicates, with 10 chicks in each of them and assigned as the following:

- Group 1B (the negative control group): was unchallenged and fed the basic diet without additives.
- Group 2A (the positive control group): was challenged with *coccidiosis* and fed on the basic diet without additives.
- Group 3A, and 4A: were challenged and fed on the basic diet supplemented with *LIP *(40g/kg of the diet), and *ANAE* (5g/kg of diet), respectively.
- Group 5A: was challenged and received the basic diet containing a combination of *LIP*, and *ANAE* at the same included levels.
- Group 3B, and 4B: were not challenged and fed on the basic diet supplemented with *LIP *(40g/kg of the diet) and *ANAE* (5g/kg of diet), respectively.
- Group 5B: was not challenged and received the basic diet containing a combination of *LIP *(40g/kg) *and ANAE* (5g/kg) at the same included levels.

### Experimental diets

Three experimental diets were formulated (Table 1) to meet the Ross broiler chicks’ nutrient requirements according to Broiler [22]. Starter diet (d 0 to 10), grower diet (d 11 to 24), and finisher diet (d 25 to 35). Representative samples of the formulated diet were taken and chemically analyzed for dry matter (DM(, crude protein) CP(, ether extract (EE), crude fiber )CF), Ash, and nitrogen-free extract (NFE (according to the method of Association of Official Analytical Chemists [23]. After using a bomb calorimeter to quantify the feed ingredients’ gross energy, metabolizable energy (ME) was calculated. Based on the feed composition tables published by Council and Nutrition [24], the percentages of methionine and lysine of the feed ingredients were computed. Diets were offered in mash *ad-libitum* daily at the morning and evening to all broilers. The fresh water was available 24 h a day during the experimental period.

**Table 1:** Ingredients and composition of the basal diet as percentage

Items	Starter diet (0–10 d)	Grower diet (11–24 d)	Finisher diet (25–35 d)
**Ingredient Composition (%)**			
Ground yellow corn	55.51	58.73	63.16
Soybean meal (46% CP)	32.52	28.70	23.58
Corn gluten meal	6.00	6.00	6.00
Sunflower oil	1.55	2.61	3.57
Dicalcium phosphate	2.00	1.77	1.59
Ground limestone	1.38	1.23	1.14
DL-Methionine	0.28	0.23	0.24
L-Lysine	0.16	0.13	0.12
Common salt	0.30	0.30	0.30
Premix^a^	0.30	0.30	0.30
**Nutrient composition (%)**			
Dry matter	84.78	85.27	85.56
Crude protein	23	21.50	19.50
Ether extract	3.79	4.91	5.95
Crude fiber	2.27	2.14	1.97
Nitrogen free extract	53.18	54.34	55.97
Ash	2.54	2.38	2.17
Calcium	0.96	0.87	0.79
Available Phosphorus	0.30	0.44	0.40
Lysine	1.31	1.20	1.12
Methionine	0.53	0.50	0.47
ME (Kcal/Kg)	3000	3100	3200

^a^Premix: HY- Mix for broilers produced by Misr Feed Additives Company, Egypt. Each 3 kg contain: Vit. A 12000.000 IU, Vit. D3 3000.000 IU, Vit. E 15.000 mg, Vit.K3 3000 mg, Vit.B1 2000 mg, Vit.B2 6000 mg, Vit.B6 3000 mg, Vit. B12 15 mg, Pantothenic acid 10.000 mg, Nicotenic acid 40.000 mg, Biotin 75 mg, Folic acid 1500 mg, Selenium 0.30 mg, Manganese 100 mg, Zinc 80 mg, Iodine 1 mg, Iron 40 mg, Copper 10 mg, Cobalt 0.15 mg and calcium carbonate (CaCO3) carrier to 3000 g

### Housing and management

The experiment was extended for 5 weeks and conducted in the experimental poultry houses which located in the Faculty of Veterinary Medicine, Aswan University, Aswan, Egypt. To avoid cross-contamination with coccidia, challenged and non-challenged birds were reared away from each other in two separate clean, well-ventilated rooms, which were fumigated and disinfected with formaldehyde gas (obtained by mixing 40% formalin and potassium permanganate powder). The proper temperature of the rooms was adjusted by using electric lamps (200 watts) in each partition, which were light and dark for 23/1 hrs/day. The ambient temperature in the experimental rooms was maintained at 34 °C during the first three days and gradually decreased by 0.5 °C daily till reached 25 °C at the end of the third week, and then it was maintained at 24 °C till the end of the experiment.

The floor of each room was divided into 12 compartments (4 treatments x 3 replicates in each room) each of 2m^2^ floor area and bedded with fresh, clean wood shavings forming 4 centimeters deep litter. Each compartment was provided with a cylindrical plastic feeder and waterer. To control diseases and increase viability, all birds were subjected to a prophylactic and pharmacological program against viral and bacterial diseases. Chicks were vaccinated against New Castle viral disease using Hitchner B1 strain (produced by *KBNP*.*INC*. Co. and has batch no. BNL0820) at the age of 8 days via eye drops. The infectious bursal disease vaccine (produced by *MEVAC Co.* and has batch no. 2208300401) was administrated to birds at the age of 14 days via eye drop. Birds were vaccinated against avian influenza (H5N1 inactivated Vaccine, produced by Zhaoqing Danhuanong Biology Medicine Co., Batch NO: 119120) at 16th day via S/C inoculation and also against New Castle virus disease using Lasota (produced by *MEVAC company* and has batch no. 2302020401) at 18^th^ day via eye drops.

### Studied parameters

#### Growth performance estimation

Broilers in each replicate were weighed as a group replicate and daily feed intake was also recorded weekly for 5 weeks of age to calculate the cumulative feed intake (FI) for each group. Body weight gain (BWG), feed conversion ratio (FCR, g feed/g gain), and performance index (PI) were calculated according to [25]. Mortality and viability of birds were monitored daily, and mortality percentage for each replicate per period was calculated. After calculation of viability % and FCR, the European Production Efficiency Factor (EPEF) and European Broiler Index (EBI) were used to evaluate the performance of broilers as suggested by Marcu et al. [26] as follows:

$$\mathbf{EPEF}\frac{\mathrm{Viability}\;\left(\%\right)\times\;\mathrm{BW}\;\left(\mathrm{kg}\right)}{\mathrm{Age}\;\left(\mathrm d\right)\times\;\mathrm{FCR}\;\left(\mathrm{kg}\;\mathrm{feed}/\;\mathrm{kg}\;\mathrm{gain}\right)}\times100$$

$$\mathbf{EBI}=\frac{\mathrm{Viability}\;\left(\%\right)\;\times\mathrm{ADG}\;\left(\mathrm g/\mathrm{chick}/\mathrm{day}\right)}{\mathrm{FCR}\;\left(\mathrm{kg}\;\mathrm{feed}/\mathrm{kg}\;\mathrm{gain}\right)\times10}$$

Where:

TWG (Total or Cumulative weight gain) = Body weight (g) at the end - Body weight (g) at start of experiment.

ADG (Average daily gain “g/chick/d”) = TWG/ days of experimental period.

FCR (Feed conversion ratio “kg feed/kg gain”) = Cumulative feed intake (kg) /Total weight gain (kg).

Viability (%) = 100 – Mortality (%).

#### Blood sampling

At the end of the experiment, three randomly selected birds from each group (one from each replicate) were selected for blood samples collection. The blood samples were collected from wing vein in non-heparinized tubes. Serum was separated by centrifugation at 3000 rpm for 10 min and stored at −18 °C till subsequent serum biochemical analysis which was measured by spectrophotometer.

##### Biochemical metrics

Serum levels of Alanine transaminase (ALT), Aspartic transaminase (AST), blood urea nitrogen (BUN), serum creatinine (CRE), total protein (TP), albumin (ALB), cholesterol, triglycerides (TG), and glucose were measured using an automatic biochemical analyzer (NEUBAO7) manufacturer’s instructions (Spectrum-Bioscience company for biotechnology, Cairo, Egypt). However, globulins were calculated mathematically by subtracting (TP -ALB).

##### Cytokine biomarkers analysis

The bioactive protein assay in the chicken serum was performed using the sandwich method of ELISA. Several biomarkers, such as tumor necrosis factor alpha (TNF-α), interleukin 10 (IL-10), and interleukin 4 (IL-4) were assessed in the serum using ELISA kits according to the manufacturer’s instructions of LSBIO Company (Cairo, Egypt). TNF-α catalog NO.LS-F23677, IL-10 catalogue NO.LS-F23347, and IL-4 catalogue NO.LS-F5318.

#### Fecal sampling

##### Identification of coccidial oocysts in excreta (total oocyst count)

To confirm the challenge model, excreta samples of both challenged birds and unchallenged controls were collected on days 6, 7, 8, 9, 10, 11, 12 and 13 after coccidial inoculation, samples were microscopically examined for the presence of oocysts to validate the challenge paradigm. Modified McMaster’s approach was used to evaluate oocyst per gram (OPG) counts [27] in feces.

$$\mathbf X=\frac{\mathrm{Total}\;\mathrm{no}.\;\mathrm{of}\;\mathrm{Oocyst}}{\mathrm{Total}\;\mathrm{no}.\;\mathrm{of}\;\mathrm{counting}\;\mathrm{chambers}}\times200$$

X = Oocyst per gram of feces.

Or X = N x 200.

N = the number of oocysts counted in one cell chamber.

#### Tissue sampling

The birds were slaughtered and their livers frozen for analysis of oxidative stress and the intestinal cecum specimens stored in 10% neutral buffered formalin for histological analysis.

##### Oxidative stress parameter

Liver samples were analyzed for superoxide dismutase (SOD) and glutathione peroxide (GPX) concentrations. Each gram of liver samples was homogenized in 5–10 milliliters of cold buffer (potassium phosphate solution, 100 mM, pH 7.0, containing 2 mM EDTA). The supernatant was separated by centrifugation at 4000 rpm for 15 min and stored at 4 °C till the subsequent analysis. While, malondialdehyde (MDA) was detected in the serum samples of birds for each group. All enzymatic assays were conducted by spectrophotometer. The commercial kits used for measures of oxidative stress parameters were purchased from Bio-Diagnostic Company (Giza, Egypt).

##### Intestinal and cecal histopathological examination

Collected specimens from small intestine and cecum were fixed in 10% neutral buffered formalin solution minimally for 24 h, dehydrated in graded ethanol, cleared in xylene, and embedded in paraffin, then sectioned at 5 μm thick tissue sections, stained with hematoxylin and eosin (H&E) and examined microscopically for any histopathological alterations [28].

##### Lesion scores

The ordinal technique is used to score the lesions. This method involves assigning data to pre-established categories groups and placing them in a “ordered” progression of lesion severity. Histologic analysis is used to provide semi quantitative ratings for lesions [29] .

All section images were taken using a Swift microscope associated with a Swift digital camera.

### Statistical analysis

Using SPSS version 14, one-way ANOVA was used to analyze the recorded data, and differences from (*P* < 0.05) were deemed significant.

## Results

### Growth performance parameters

As shown in Table 2, the growth performance parameters of broilers are significantly (*P* > 0.05) affected by the inclusion of *LIP*, *ANAE*, and a combination from *LIP* + *ANAE* in their diets. From d 0 to d 35, positive control broilers had lower final BW, cumulative WG, ADG and poorer FCR compared to negative control and the all other challenged and non-challenged broilers (*P* < 0.05). Non-challenged broilers fed on *LIP*, *ANAE* alone and in combination had greater final BW, total & daily WG and cumulative FI as well as had better FCR compared to challenged broilers given the same additives. Challenge x additive interactions were observed for BW, WG and FCR as well as for total FI (*P* < 0.05). The interactions indicated that adding of *LIP*, *ANAE* or mixture from both additives exerted a greater positive effect on the final BW, cumulative WG, ADG, FCR and total FI of Eimeria challenged broilers compared to positive control. All challenged broilers had lower PI, EPEF, and EBI (*P* < 0.05) compared to non-challenged broilers. Also, the lowest values were recorded in positive control group in comparison with negative control. In general, the best results of overall growth performance parameters of challenged and non-challenged broilers were recorded in a group fed on mixed additives (*LIP* + *ANAE*) followed by group fed on *ANAE* and lastly group fed on *LIP* in comparison with control.

**Table 2:** Growth performance parameters of broiler chickens during the experimental period

Groups^**^	1	2	3 A	4 A	5 A	3B	4B	5B
Parameters								
**Initial B.W. (g)**	45.5±0.84	45.6±0.65	45.14±0.74	44.72±0.81	43.49±0.79	44.6±0.6	45.38±0.7	43.05±0.91
**Final B.W. (g)**	1970.26±45.19^ab*^	1743.83±46.62^d^	1806.89±46.33^c^	1887.22±45.42^bc^	1901.34±46.11^b^	2002.41±46.72^a^	2080.63±45.39^a^	2122.80±45.81^a^
**Cumulative W.G. (g)**	1924.75± 46.31^ab^	1698.25± 39.89^c^	1761.75± 53.27^bc^	1842.50± 50.64^b^	1857.85± 45.55^b^	1957.78± 42.21^ab^	2035.25± 51.47^a^	2079.75± 47.11^a^
**ADG (g)**	54.99± 4.62	48.52± 4.81	50.34± 3.45	52.64± 4.21	53.08± 5.11	55.94± 3.87	58.15± 4.76	59.42± 5.39
**Cumulative F.I. (g)**	3262.13± 76.23^b^	3052.± 77.13^c^	3127.74± 66.76^bc^	3139.13± 74.39^bc^	3168.48± 68.57^bc^	3228.23± 64.87^b^	3389.99± 71.56^ab^	3419.17± 69.94^a^
FCR **(g F.I./ g W.G.)**	1.69± 0.16^b^	1.80± 0.02^a^	1.78± 0.14^ab^	1.70± 0.13^ab^	1.71± 0.04^ab^	1.65± 0.11^b^	1.67± 0.06^b^	1.64± 0.08^b^
**PI (%)**	116.58± 3.44^ab^	96.88± 4.57^b^	101.51± 4.36^ab^	111.01± 3.91^ab^	111.19± 3.77^ab^	121.36± 2.98^a^	124.59± 4.16^a^	129.44± 3.47^a^
**EPEF**	333.10± 15.13^a^	221.4± 15.33^c^	261.03± 14.77^b^	285.46± 14.59^b^	307.20± 16.88^ab^	346.74± 16.49^a^	355.97± 15.87^a^	369.83± 17.65^a^
**EBI**	325.38± 15.56^a^	215.6± 14.11^c^	254.53± 15.62^b^	278.68± 16.32^b^	300.17± 17.51^ab^	339.03± 16.29^a^	348.20± 17.46^a^	362.32± 19.55^a^

When data were expressed as mean ± SME (standard error of the means), the symbol * → was used. Using different letters to indicate statistical significance. The means within the same raw with different superscripts are substantially different (*p* < 0.05), as indicated by the highest significance a, ab, b, bc, and c →

Table 3 shows that no mortality rates were recorded in treated unchallenged groups and negative control. While, challenged broilers fed on diets containing *LIP* or *ANAE* accompanied by 10% of mortality for each followed by challenged group fed on diet supplemented with a mixture from two additives (3.3%) compared to positive untreated control group which recorded the highest mortality rate (20%). Present results indicated that feeding of broilers on diets supplemented with *LIP*, *ANAE* alone or mixture from both additives could be helped in decreasing the mortality rates which caused by coccidiosis.

**Table 3:** Mortality rate of broilers chickens during the experimental period

Groups	1	2	3 A	4 A	5 A	3B	4B	5B
Weeks								
Total No.	30	30	30	30	30	30	30	30
Dead No.	0	6	3	3	1	0	0	0
Survival rate %	100	80	90	90	96.7	100	100	100
Mortality rate %	0	20	10	10	3.3	0	0	0

### Biochemical parameters

**Fig. 1:**
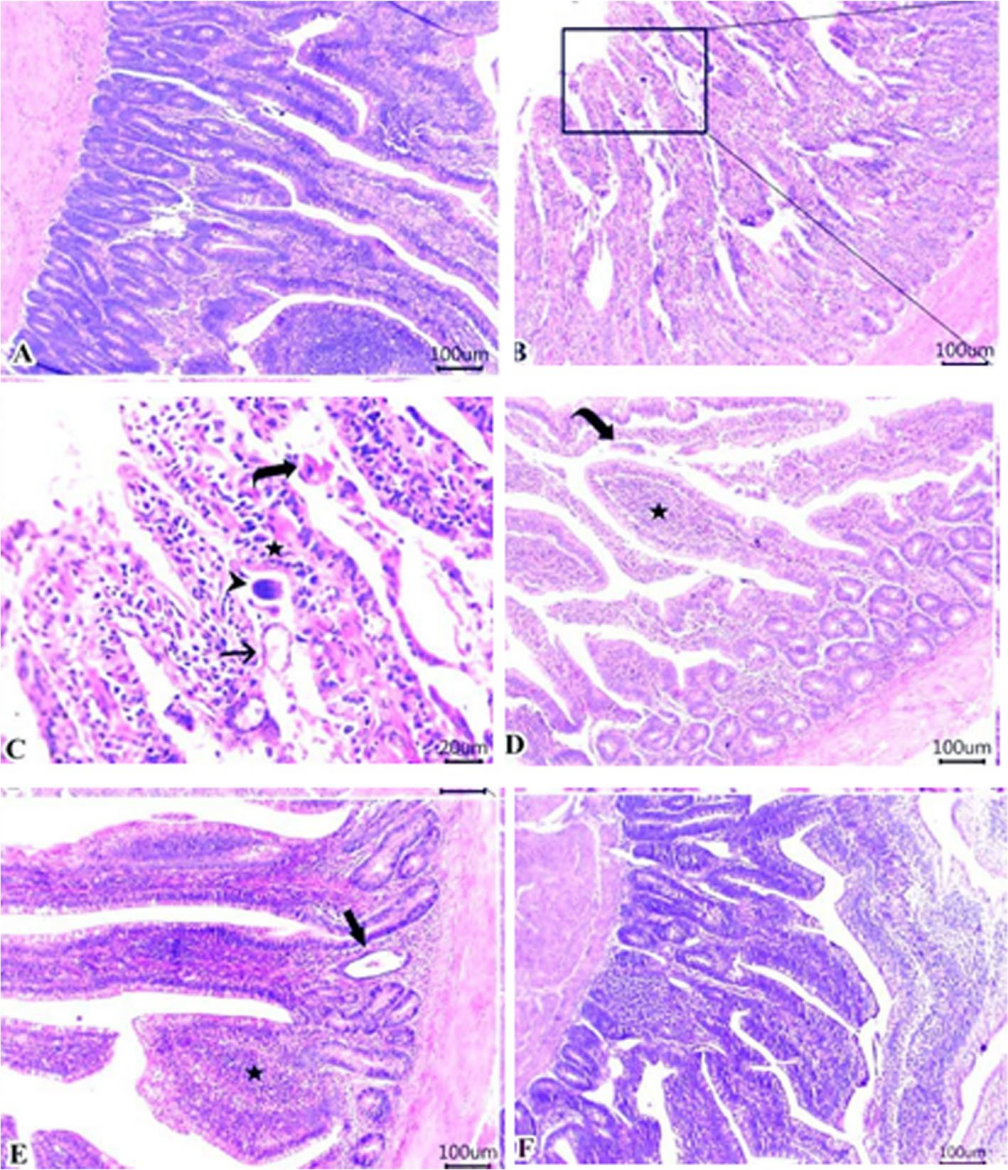
Evaluation Of MDA.** a **showing normal villous epithelium, crypts, lamina propria, submucosa and muscular is mucosa. **b**, **c **showing necrotic, detached epithelial lining villi (curved arrow) and numerous developmental stages of coccidea “macrogamete (arrow) & microgamete (arrowhead)” beside numerous inflammatory cells infiltration particularly lymphocytes (star) within the lamina propria & submucosa. **d **showing detached epithelium (curved arrow) and impacted villous core with lymphocytes, eosinophils and extravasated erythrocytes (star).** e **showing cystic dilatation of some intestinal crypts (thick arrow) and mild inflammatory cells infiltrates within lamina propria and submucosa. **f** showing intact intestinal architectures of mucosa, lamina propria, submucosa and muscular layer

**Fig. 2:**
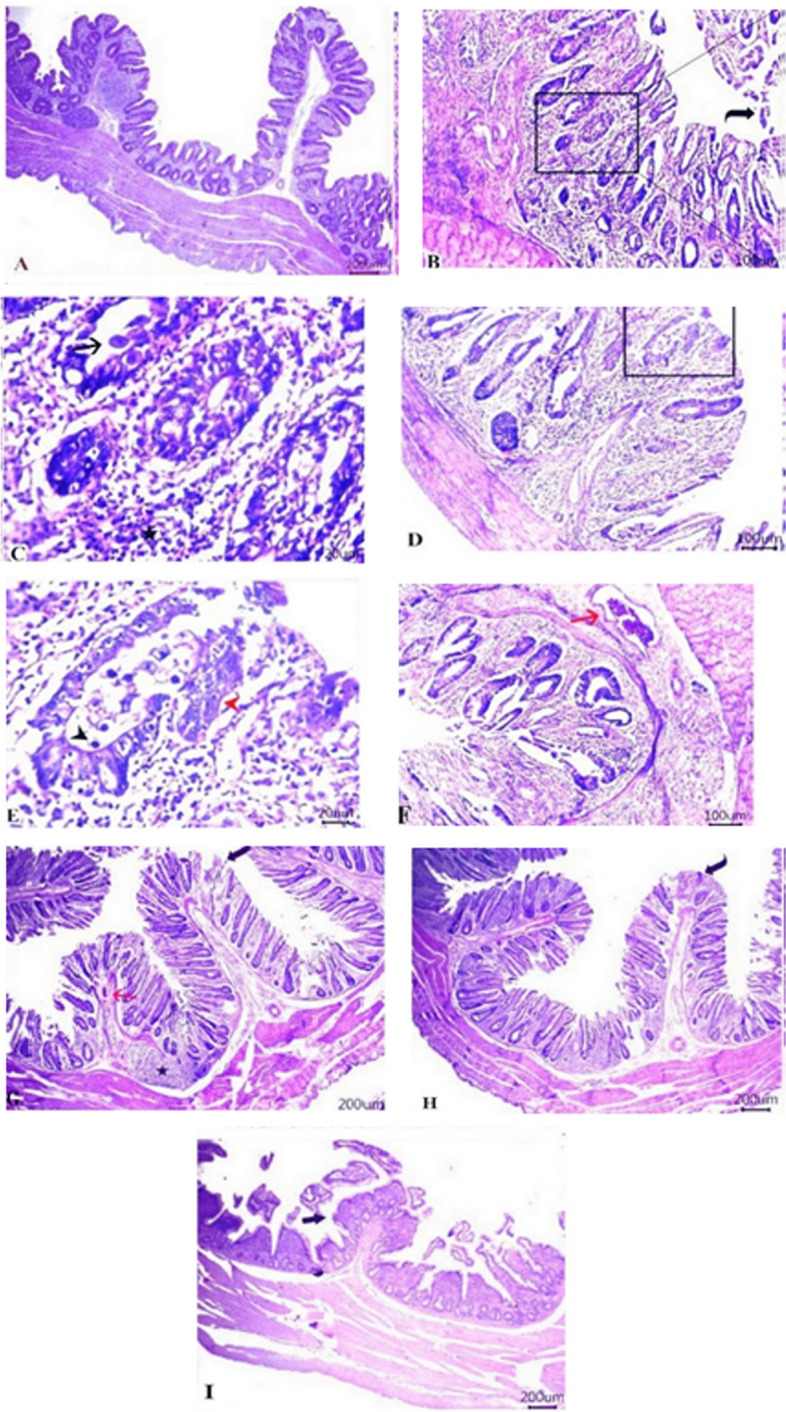
Evaluation Of SOD. **a** showing normal histological architectures of epithelial lining mucosa, submucosal layer, muscularis and serosa. Infected group with coccidia. **b**, **c** showing necrotic mucosal epithelium (curved arrow), abundant inflammatory cells infiltrates in between destructed crypts (star), occupied large number of mucosal epithelium & crypts by developmental stages of coccidian. **d**, **e** showing aggregation of oocytes (black arrowhead) adhered to hyperplastic mucosal epithelium (red arrowhead). **f** showing cystic cecal crypts with remnants of mucous secretions. **g** showing focal necrotic area of mucosal epithelium (curved arrow), destructed crypts, dilated submucosal vasculatures (red arrow), and prominent (GALT) within submucosal layer (star). **h** treated coccidea group by Acacia showing necrotic some mucosal epithelium (curved arrow). **i** showing numerous goblet cells within epithelial lining mucosa (black thick arrow)

**Fig. 3:**
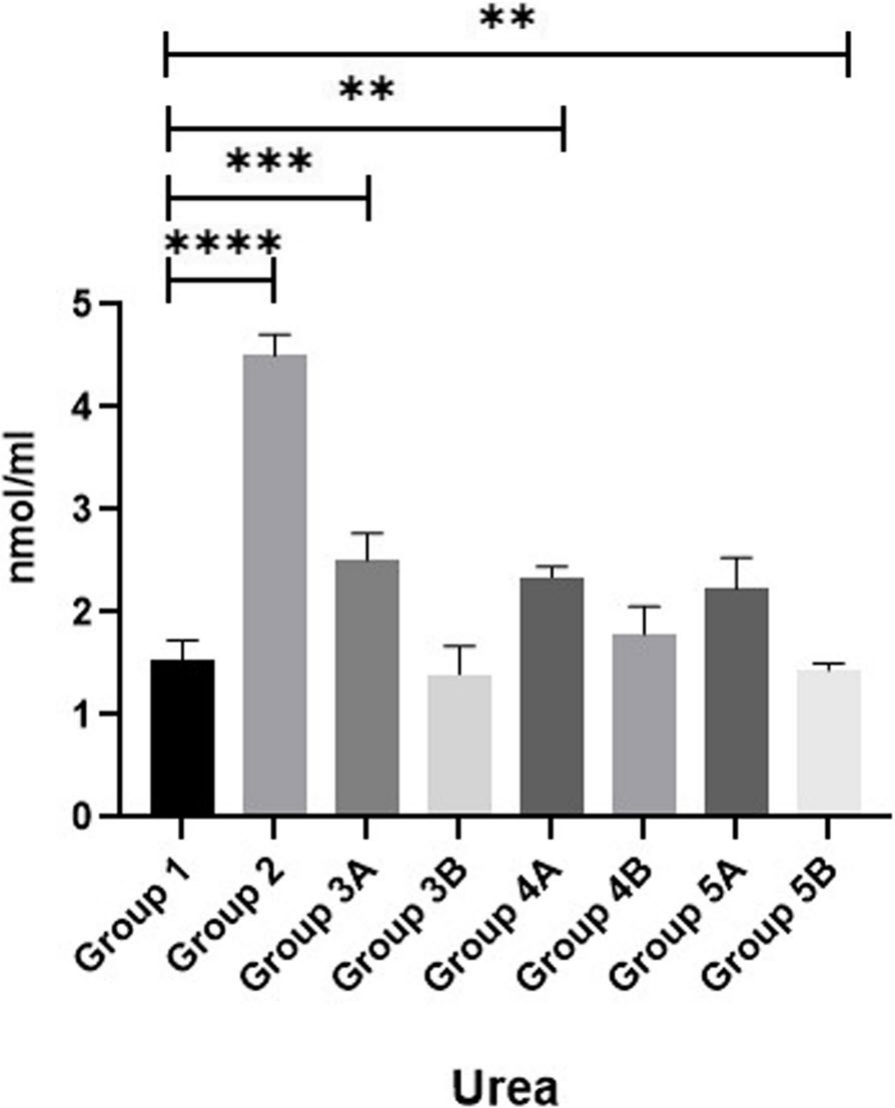
Evaluation of GSHPX

**Fig. 4:**
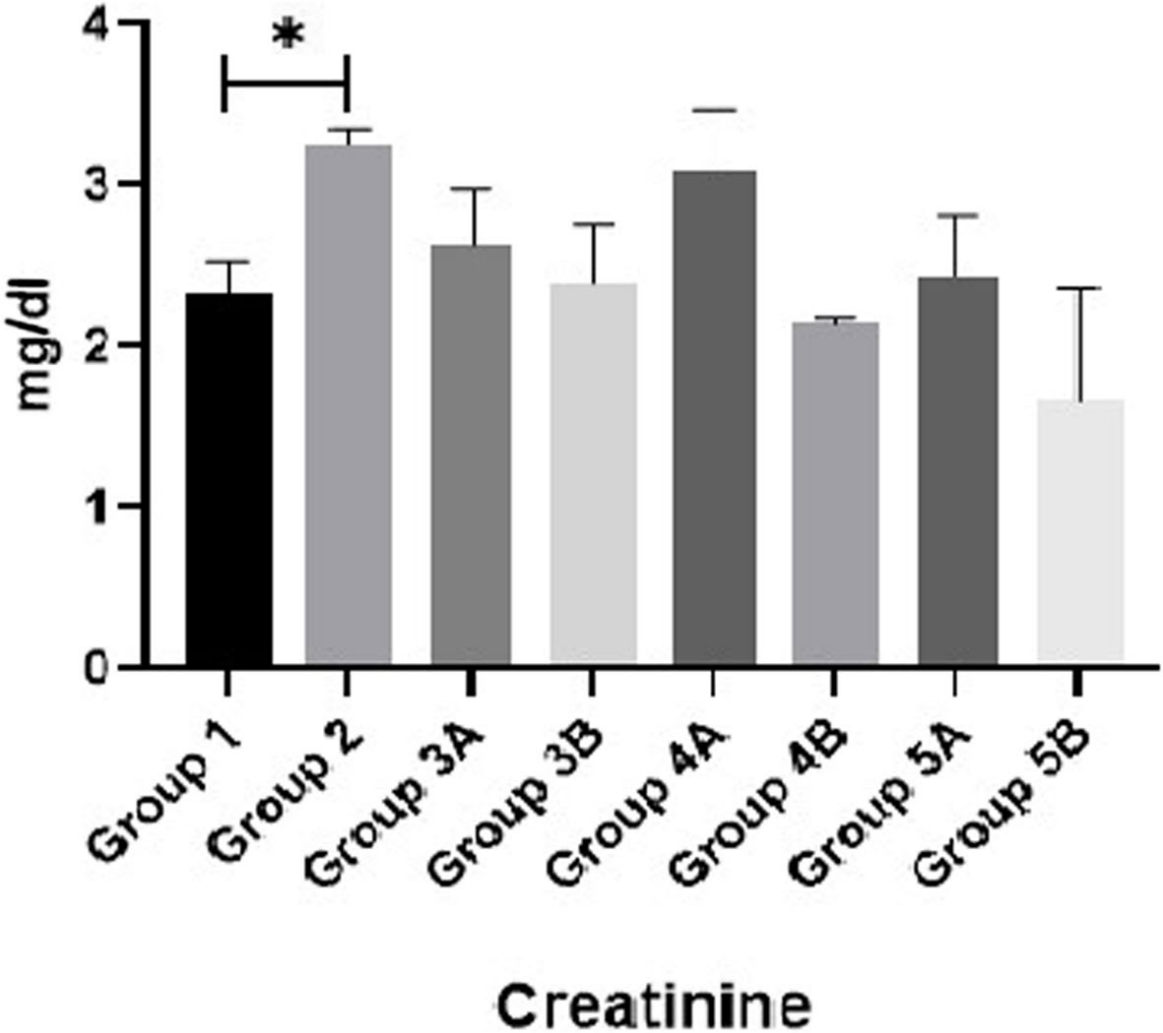
Evaluation of ALT

**Fig. 5:**
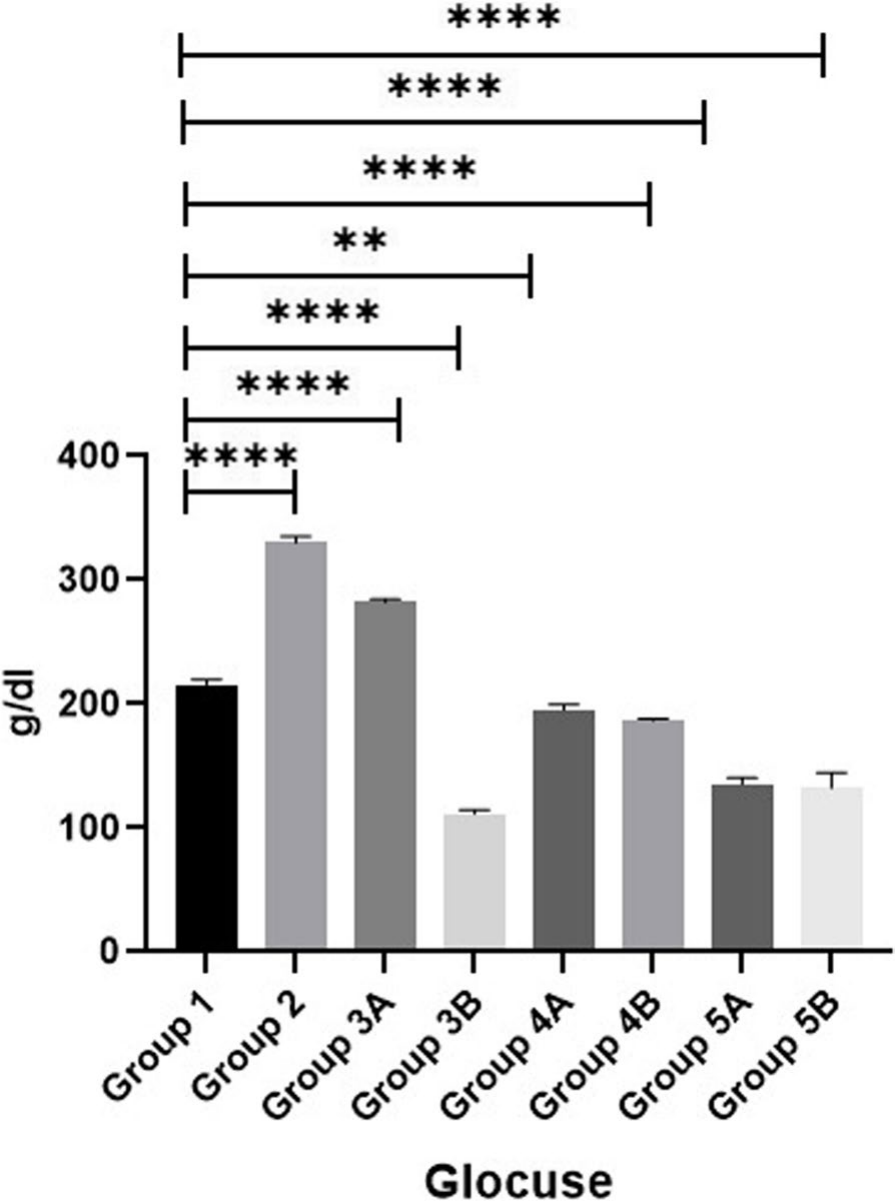
Evaluation of AST

 The results of serum liver function tests (AST & ALT), kidney function tests (BUN & CRE) and glucose are shown in Figs. 1, 2, 3, 4 & 5. Data demonstrated that all challenged broilers had higher AST, ALT, BUN, CRE& glucose levels compared to all non-challenged broilers. Also, the highest values were recorded in positive control group (G2) in comparison with negative control group (G1). By addition of *LIP* and *ANAE* alone or mixture from them in the diets of challenged broilers led to decrease in the liver & kidney function tests and serum glucose in comparison with positive control group (G2). Therefore, the lowest values were recorded in challenged birds fed on diet supplemented with a mixture of *LIP* and *ANAE* (G5A) followed by challenged birds fed on diet containing *ANAE* (G4A) and *LIP* (G3A), respectively compared with positive control group (G2). Similarly, in non-challenged groups, lowest values were achieved in G5B followed by G4B and G3B, respectively compared with negative control group (G1).

**Fig. 6:**
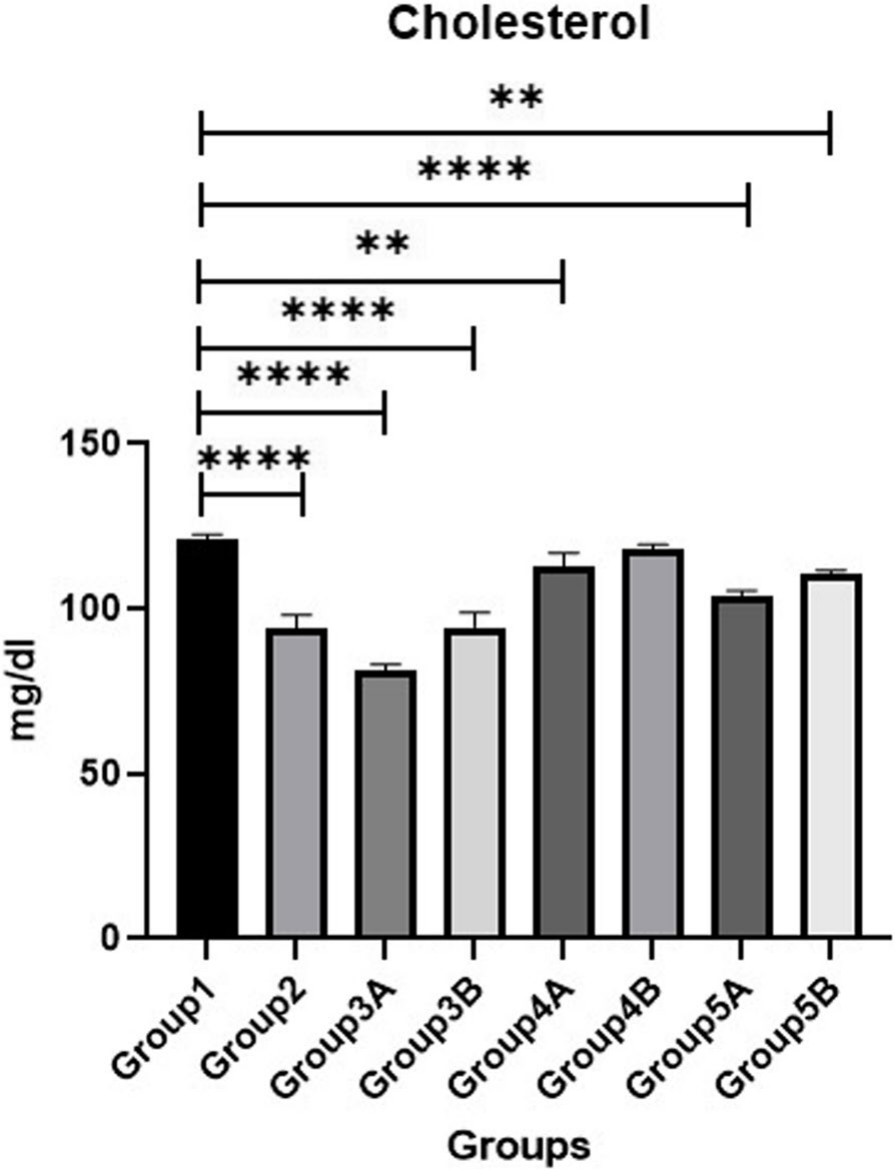
Evaluation of albumin

**Fig. 7:**
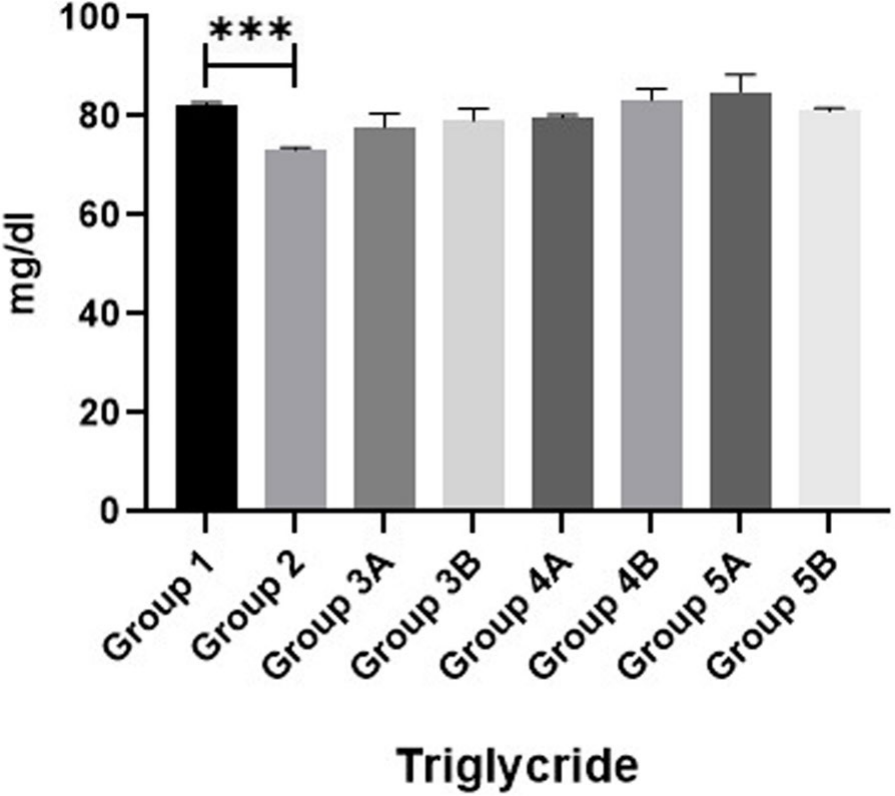
Evaluation of total protein

 Data concerning lipid profiles (cholesterol and triglycerides) of broilers are shown in Figs. 6 & 7. The concentrations of serum cholesterol and triglycerides in all challenged groups were lower than all non-challenged groups. The lowest values were recorded in positive control group (G2) in comparison with negative control group (G1). Addition of *LIP* and *ANAE* alone or in combination to the diets of challenged broilers led to increase in the serum cholesterol and triglycerides in comparison with positive control group (G2). Consequently, the highest values were recorded in challenged birds fed on diet supplemented with a mixture of *LIP* and *ANAE* (G5A) followed by challenged birds fed on diet containing *LIP* (G3A) *and ANAE* (G4A), respectively compared with positive control group (G2). By comparison between non-challenged groups, lowest values were achieved in G5B followed by G4B and G3B, respectively compared with G1 (negative control group).

**Fig. 8:**
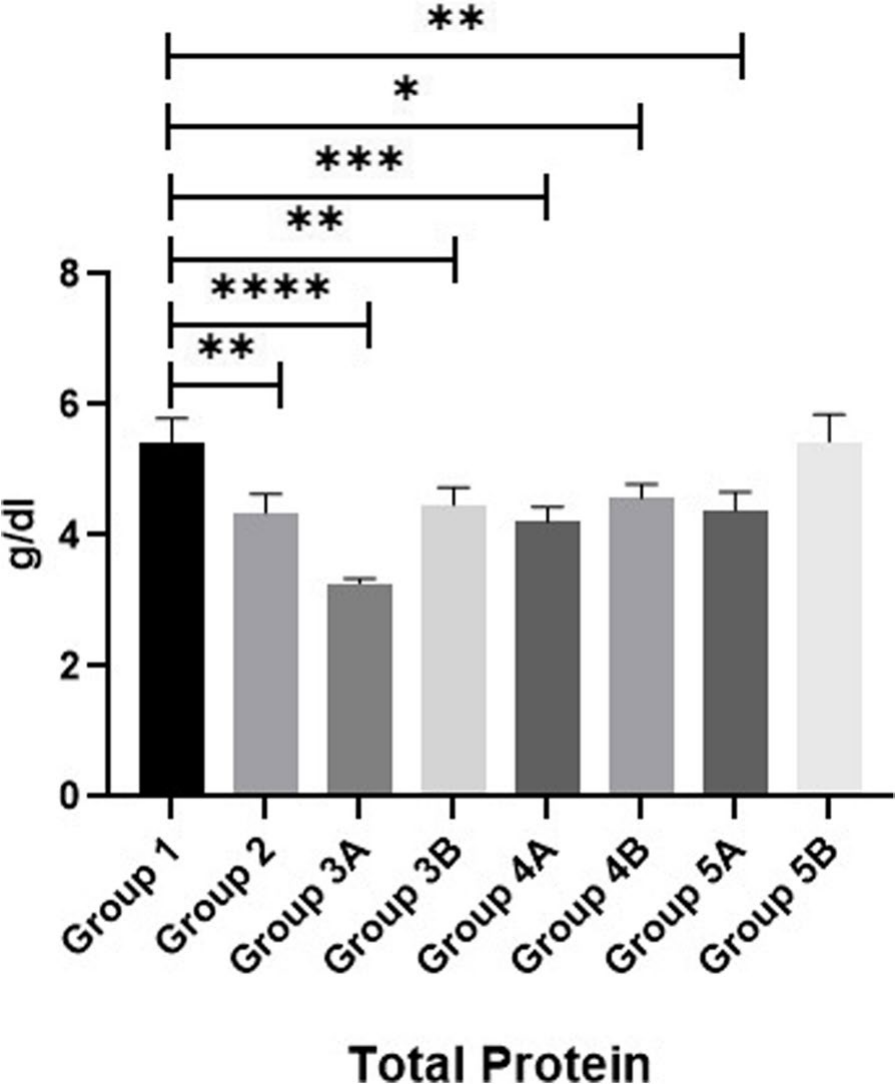
Evaluation of globulin

**Fig. 9:**
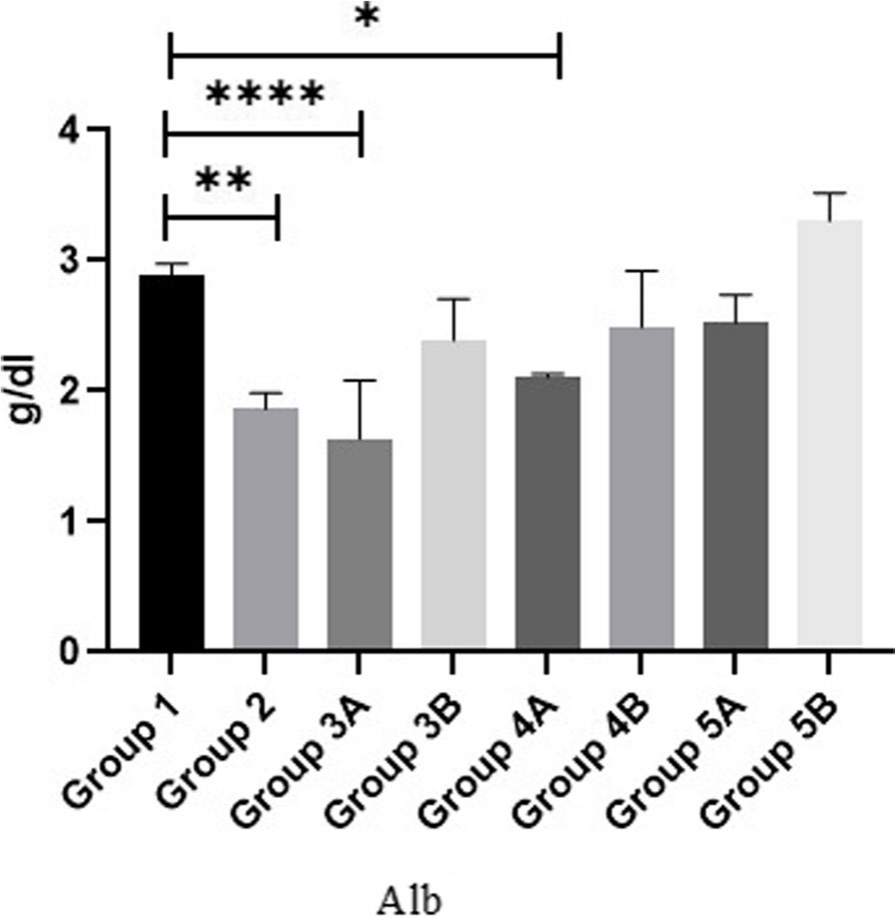
Evaluation of glucose

**Fig. 10:**
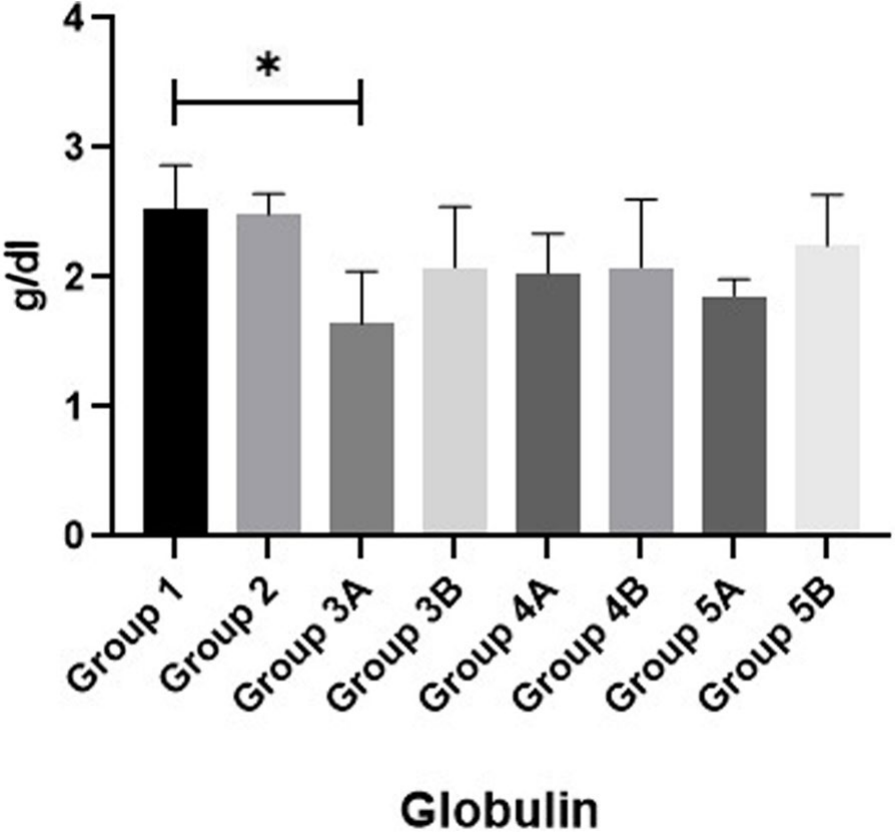
Evaluation of cholesterol

 Regarding the serum total protein (TP), albumin (ALB) and globulin of broilers, results in Figs. 8, 9 & 10 showed that all non-challenged broilers had higher TP, ALB& globulin levels compared to all challenged broilers. The lowest values were recorded in positive control group (G2) in comparison with negative control group (G1). Supplementation of *LIP* and *ANAE* alone or mixture from them in the diets of challenged broilers led to decrease in the serum total protein and its fractions in comparison with positive control group (G2). Moreover, the lowest values were recorded in challenged birds fed on diet supplemented with *LIP* (G3A) followed by challenged birds fed on diet containing *ANAE* (G4A) and a mixture of *LIP* and *ANAE* (G5A), respectively compared with positive control group (G2). By comparison between non-challenged groups, lowest values were achieved in G3B followed by G4B and G5B, respectively compared with negative control group (G1).

### Oxidative stress parameters

**Fig. 11:**
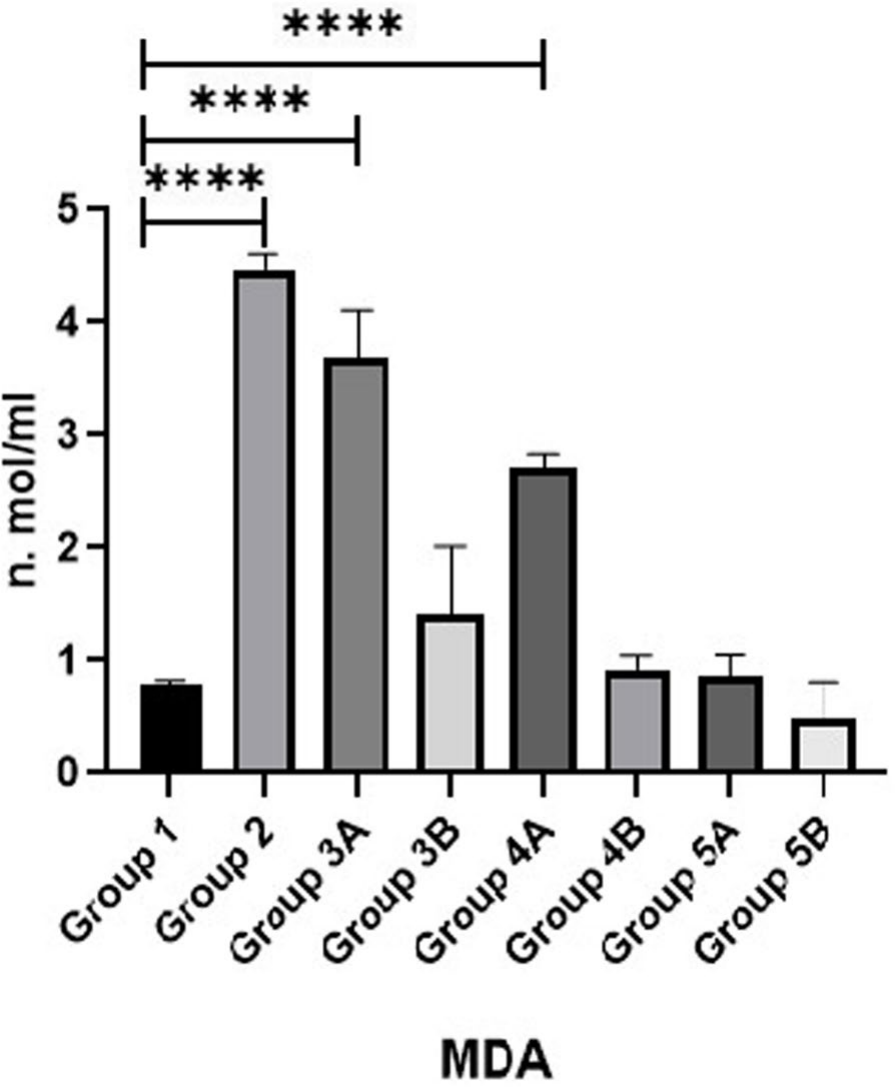
Evaluation of triglycerides

**Fig. 12:**
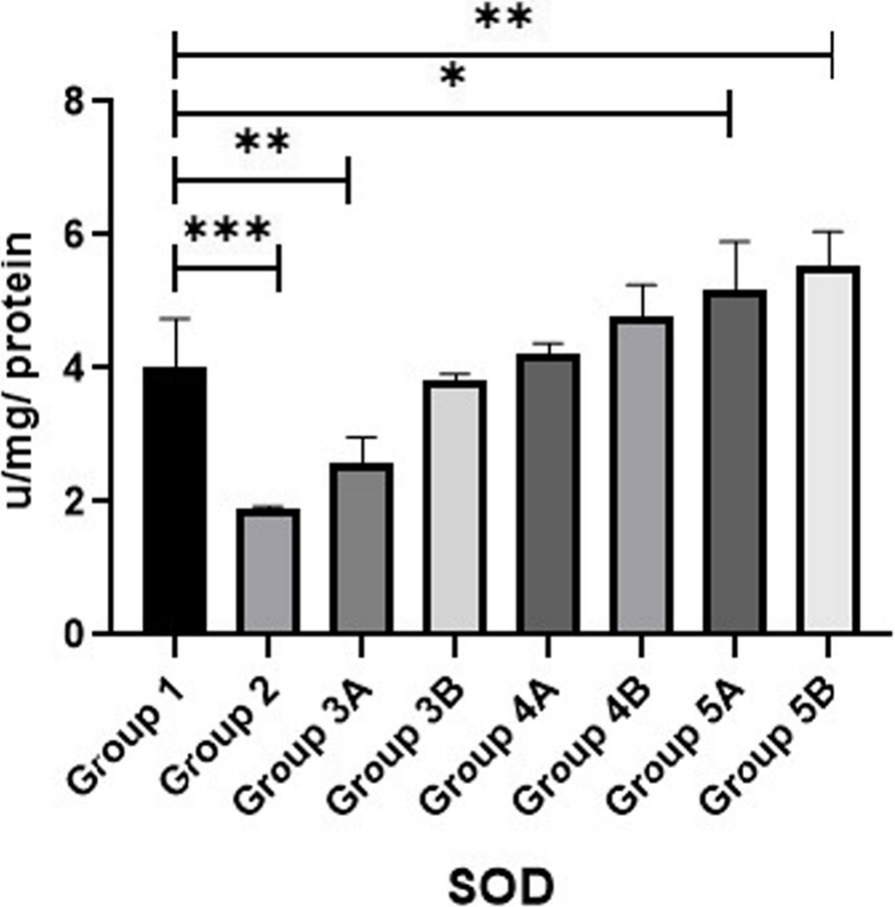
Evaluation of urea

**Fig. 13:**
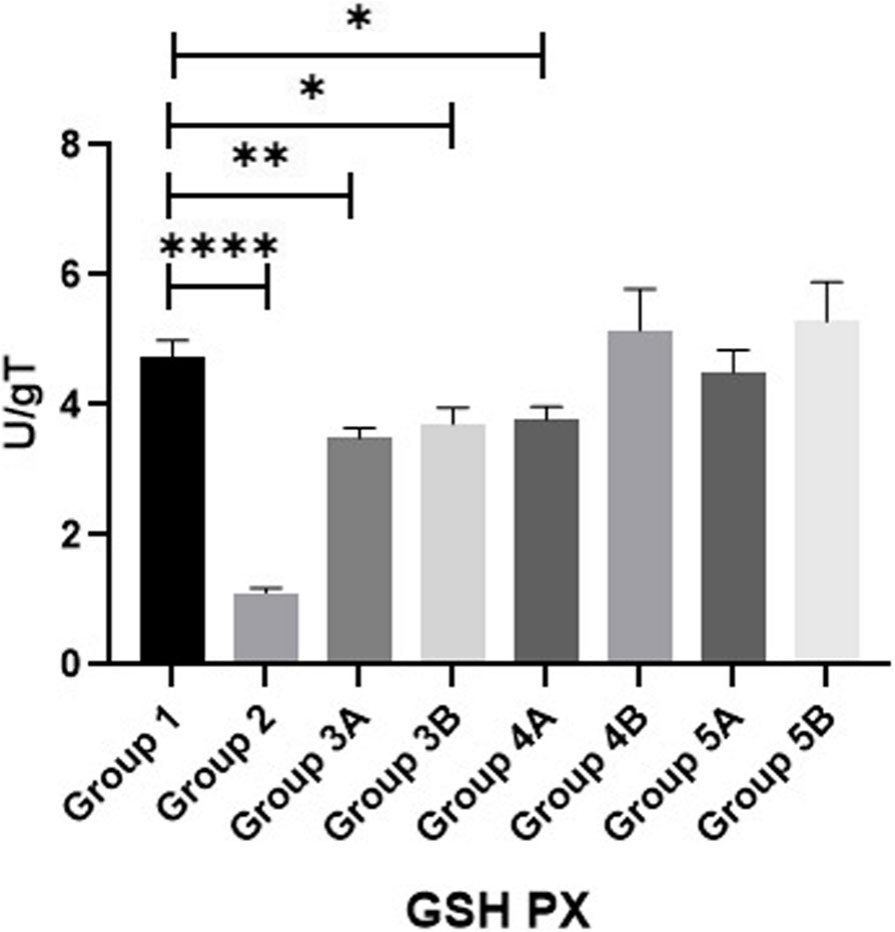
Evaluation of creatinine

 Results of MDA, SOD and GPX are shown in Figs. 11, 12 & 13. Data showed that all challenged broilers had higher MDA and lower SOD & GPX levels compared to all non-challenged broilers. Additionally, the highest MDA value and the lowest SOD & GPX were recorded in positive control group (G2) in comparison with negative control group (G1). Inclusion of *LIP* and *ANAE* alone or mixture from them in the diets of challenged broilers with coccidia led to decrease in serum MDA and increase in both SOD & GPX in comparison with positive control group (G2). Therefore, lowest MDA were recorded in challenged birds fed on diet supplemented with a mixture of *LIP* and *ANAE* (G5A) followed by challenged birds fed on diet containing *ANAE* (G4A) and *LIP* (G3A), respectively compared with positive control group (G2). While, the highest SOD and GPX were recorded in G5A followed by G4A and G3A, respectively compared with G2. By comparison between non-challenged groups, the lowest MDA and the highest SOD & GPX values were achieved in birds fed on diet containing a mixture of *LIP* and *ANAE* (G5B) followed by birds fed on diet supplemented with *ANAE* (G4B), while the highest MDA and the lowest SOD & GPX values were recorded in birds fed on *LIP* (G3B) in comparison with G1.

### Cytokine biomarkers

**Fig. 14:**
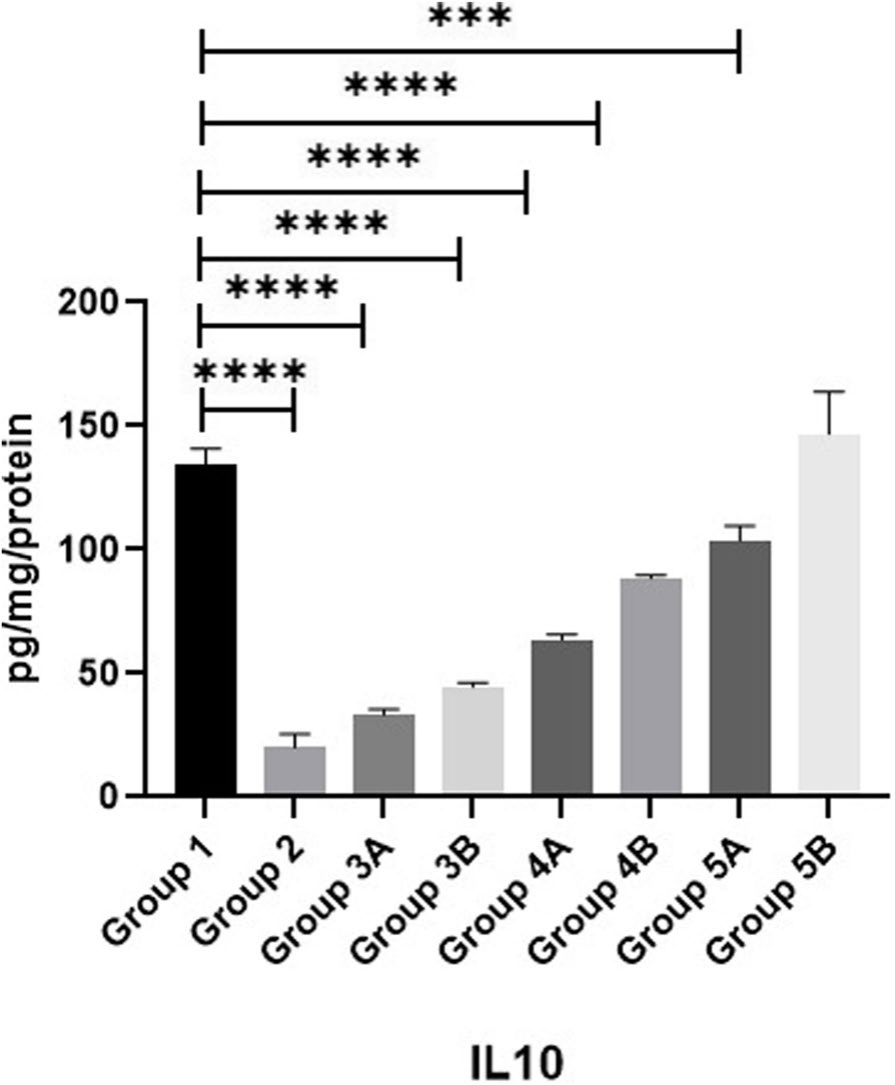
Evaluation of IL10

**Fig. 15:**
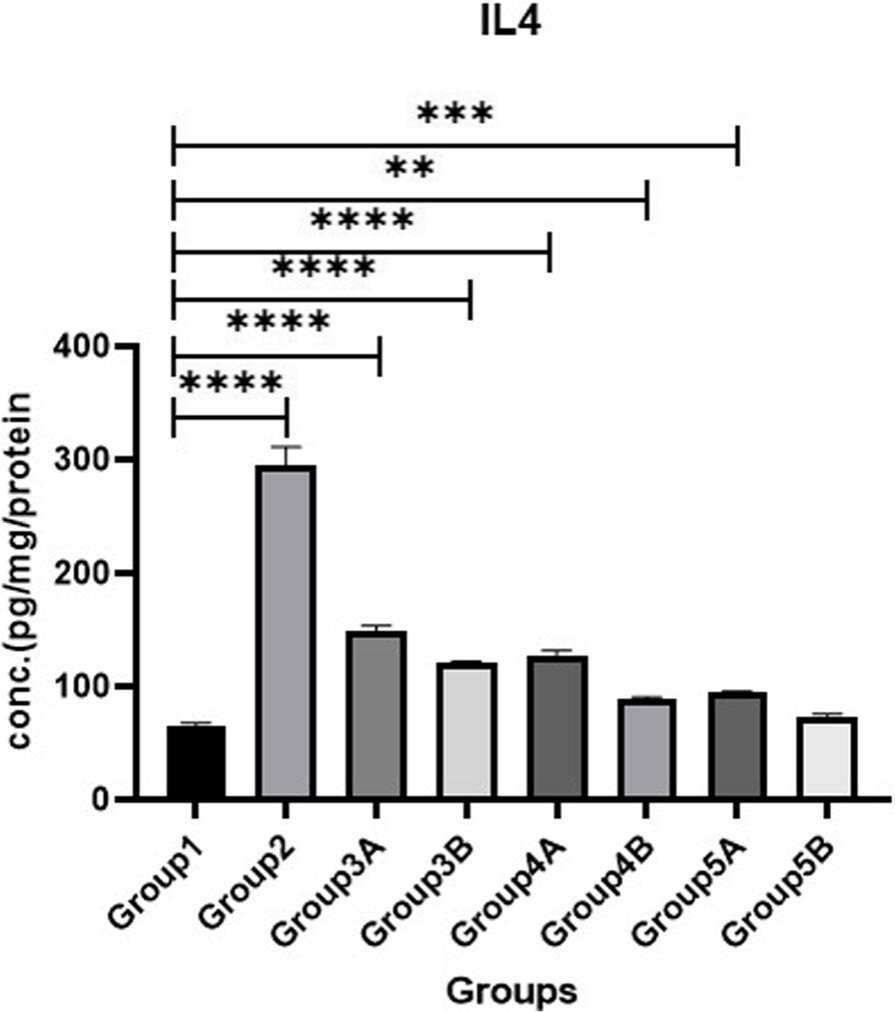
Evaluation of αTNF

**Fig. 16:**
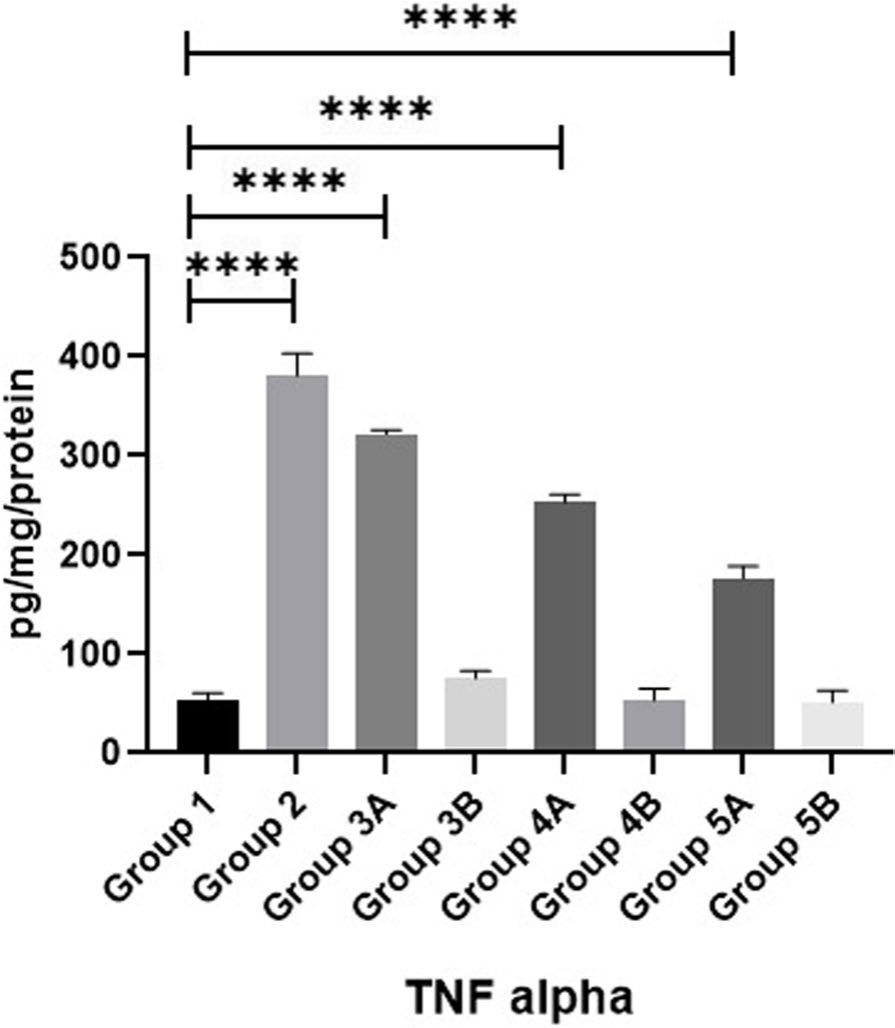
Evaluation of IL4

 Measuring IL-10, IL-4 & TNF-α as illustrated in Figs. 14, 15 & 16 revealed that all challenged groups had lower IL-10 and higher IL-4 & TNF-α than all non-challenged groups. The lowest IL-10 and the highest IL-4 & TNF-α were recorded in positive control group (G2) in comparison with negative control group (G1). Supplementation of *LIP* and *ANAE* alone or in combination to the diets of challenged broilers led to increase in IL-10 and decrease in IL-4 & TNF-α compared with positive control group (G2). Therefore, the highest IL-10 and the lowest IL-4 & TNF-α were recorded in challenged birds fed on diet supplemented with a mixture of *LIP* and *ANAE* (G5A) followed by challenged birds fed on diet containing *LIP* (G3A) *and ANAE* (G4A), respectively compared with positive control group (G2). By comparison between non-challenged groups, the highest IL-10 and the lowest IL-4 & TNF-αwere in G5B, while the lowest IL-10 and the highest IL-4 & TNF-α were in G3B followed by G4B in comparison with G1 (negative control group).

### Oocyst shedding (total oocyst count)

Both the treatments whether *LIP* only or *LIP* with *ANAE*, resulted in a significant decrease in the oocyst count on the sixth day following exposure to coccidia, as illustrated in Table 4. Comparing *LIP* and *ANAE* treatments separately to the positive control group on the seventh day revealed a significant decrease in oocyst count. Supplementation of broilers with *LIP* alone and mixed *LIP* + *ANAE* significantly decreased the oocyst count in comparison with the positive control group at the eighth day. Furthermore, when compared to the control positive group, *ANAE* alone and mixed *LIP* + *ANAE* showed a noticeably lower oocyst count on day 9 and 10, followed by *LIP* alone. In addition, the mixed *LIP* + *ANAE* showed a notable decrease in oocyst count on the eleventh day; nevertheless, the *LIP* and *ANAE* groups showed the same mean in oocyst count compared with the positive control group. Additionally, when compared to the positive control group on days 12 and 13, the mixed *LIP* + *ANAE* showed a more notable drop in oocyst count, followed by *ANAE* and then *LIP* groups.

**Table 4:** Oocyst count

Parameters	Coccidia -ve control	Coccidia + ve control	Lawsonia + coccidia	Acacia + coccidia	Combination between Lawsonia and Acacia + coccidia
Day 6	0	57.9±1.4^a^	48.4±2.2^b^	57.5±1.4^a^	54.1±4.6^a^
Day 7	0	146.4±1.3^ab^	120.2±1.3^b^	124.2±1.2^b^	156.7±1.3^a^
Day 8	0	342.4±6.6^a^	268.5±3.9^b^	288.6±47.2^ab^	269.9±7.1^b^
Day 9	0	335.2±13.4^a^	279.8±1.2^ab^	256.0±4.9^b^	261.4±9.9^b^
Day 10	0	393.4±3.4^a^	214.9±7.5^b^	199.1±1.8^c^	191.9±1.3^c^
Day 11	0	381.4±6.3^a^	169.7±1.8^b^	160.4±16.5^b^	116.1±8.1^c^
Day 12	0	293.2±5.8^a^	129.4±1.8^b^	84.3±2.5^c^	64.6±2.4^d^
Day 13	0	361.8±4.5^a^	126.9±4.3^b^	75.9±2.9^c^	58.9±4.6^d^

Using different letters to indicate statistical significance The means within the same raw with different superscripts are substantially different (p < 0.05), as indicated by the highest significance, a, b, c and d →

### Intestinal and cecal histopathology

Intestinal sections of G1B (negative control group) showed normally organized intestinal layers, including lamina propria, crypts, submucosa, muscular, and villous epithelium as shown in Fig. 1A. While, in G2A (positive control group) displayed multiple developmental stages of coccidia “macrogamete (arrow) & microgamete (arrowhead)” in addition to a large infiltration of inflammatory cells, especially lymphocytes (star) within the lamina propria and submucosa as illustrated in Fig. 1B & C. Intestinal sections of G3A treated with *LIP* showed an impacted villous core with detached epithelium (curved arrow) and extravasated erythrocytes, eosinophils and lymphocytes (star) as shown in Fig. 1D. While, Fig. 1E represented the group treated coccidia group by *ANAE* (G4A) in which the star pointing to a slight infiltration of inflammatory cells into the lamina propria and submucosa. In addition to, the thick arrow pointing to the dilatation of some intestinal crypts as a result of cystic fibrosis. Combined treated group by *LIP*& *ANAE* (G5A) showed the intact intestinal architectures of mucosa, lamina propria, submucosa and muscular layer as shown in Fig. 1F.

 Cecal sections of group 1B (negative control group) revealed intact epithelial lining mucosa, submucosal layer, muscularis mucosa and serosa as illustrated in Fig. 2A. While, G2A (positive control group) in Fig. 2B, C, D, E & F exhibited necrotic mucosal epithelium and abundant inflammatory cells infiltrates primarily lymphocytes in between destructed crypts within lamina propria. Large number of mucosal epithelium & crypts were occupied by developmental stages of coccidia particularly gametogony stages both macrogametes and microgametes. As well, aggregation of oocytes was seen adhered to hyperplastic mucosal epithelium. Cystic cecal crypts with remnants of mucous secretions were also seen. Some sections showed congestion of some submucosal vasculatures and lymphocytosis in other vasculatures. Extravasated erythrocytes between cecal crypts were also seen. Cecal sections of *LIP* treated group (G3A) showed focal necrotic area of mucosal epithelium, destructed crypts, dilated submucosal vasculatures, and prominent (GALT) within submucosal layer as shown in Fig. 2G. At the same time, treated coccidia by *ANAE* (G4A) showed maintain structures of the majority of intestinal layers with necrosis of some mucosal epithelium (Fig. 2H). While combination of *LIP*& *ANAE* (G5A) showed enhanced restoration of most structures of cecal layers with numerous goblet cells within epithelial lining mucosa (Fig. 2I).

### Lesions score of small intestine & cecal tissues among different experimental groups

Histopathological scoring lesions as illustrated in Table 5 were classified into Absent lesion (-= 0), mild ( + = 5–25%), moderate (++ = 26–50%), and severe (+++ = ˃50%) depending on the severity of the changes. Accordingly, the control negative group manifested normal architecture of the intestinal tissues. Whereas, the infected group with coccidia exhibited severe damage and necrosis of intestine indicating various stages of coccidia. Since, the intestinal tissues suffered from severe hemorrhage, inflammation and extensive congestion. Contrary to the treated groups either with *Lawsania inermis* or *Acacia Nilotica* lead to reduce the histological alterations of the intestine which developed by coccidia. At the same time, mixed group of *Lawsania inermis* and *Acacia Nilotica* displayed intact histomorphology close to those of the control negative group.

**Table 5:** Lesions score of the severity extent within small intestine & cecal tissues among different experimental groups

Lesions		G1	G2	G3A	G4A	G5A
Small intestine	Necrotic & detached mucosal epithelium.	-	+++	+	+	-
	Numerous stages of coccidian.	-	+++	+	+	-
	Inflammatory cells infiltration.	-	+++	++	+	+
	Dilatation of crypts.	-	++	+	+	-
Cecum	Necrosis & desquamated epithelium.	-	+++	+	+	-
	Congested blood vessels.	-	++	+	-	-
	Hemorrhages.	-	+	+	-	-
	Lymphocytes aggregations.	-	+++	++	+	+
	Developmental stages of coccidian.	-	+++	++	+	+

Examined chickens = 10 chickens /group. Number of examined fields (five fields/chicken, X100). The lesions were graded by estimating the percentage area affected in the entire section: Lesions score system was as follows: - = absence of lesion, + = 5–25%, ++ = 26–50%, and +++ = ˃50%.

## Discussion

### Broilers’ performance

Gut health and high productivity are intimately linked to the growth and health of meat-type chickens. It affects the broilers’ ability to effectively metabolize, absorb, and use their feed, particularly with regard to caloric and protein, as well as their resilience to disease [30]. In light of this, researchers looked at natural substances every day as possible anti-coccidiosis drugs [31]. According to Kadykalo et al. [32] and Abebe and Gugsa [33] coccidian illnesses are linked to decreased weight gain and a poor feed conversion ratio. Comparing unchallenged and challenged broilers to negative and positive controls, respectively, our results indicated that the addition of herbals and their extracts could increase body weight, weight gain and feed conversion ratio (FCR). The beneficial impact of *Lawsonia inermi*s (*LIP*) on broilers performance is consistent with the findings obtained by Soliman and Hassan [34] who observed that an improvement (*P* < 0.05) in the FCR, daily weight gain, total weight gain and final weight of broiler birds by inclusion of *LIP* in their feed. This could be as a result of *Lawsonia inermis* direct replenishment of vitamin and mineral deficits as well as its defense of the birds against many detrimental environmental stressors. Furthermore Kiavandani et al. [35] reported that an increase in the body weight and weight gain of broilers could be due to the increase of beneficial *lactobacillus* count (commensal microflora) in the gut of birds with improved nutrient utilization caused by the addition of *LIP* in broilers diets. The beneficial effects of *Acacia nilotica* aqueous extract (*ANAE*) on growth performance metrics in our study are consistent with those of Daud et al. [36] and Iqbal et al. [37] who noted that supplementing broilers with *ANAE* increased their total weight gain and FCR when compared to the control group. Also, El-Galil et al. [38] found that addition of *Acacia nilotica* leaf meal to broiler diets increased feed efficiency, FCR, weight gain and outperformed the control group. This experiment’s enhanced growth performance and FCR may have been caused by the bioactive components in *ANAE* which stimulate the production of digestive enzymes and improve the digestibility of nutrients, as previously discussed by Zahid et al. [39]. A combination of *LIP* and *ANAE* supported growth-related factors, according to Marimuthu and D’Souza [40]. Conversely, Nakalebe [41] reported that broiler feed intake was not significantly affected by *Acacia nilotica* leaf extract supplementation. While EL-Khier et al. [42] and Tabidi and Ekram [43] showed that supplementing of broiler chickens with *Acacia Senegal* increased feed intake but had no discernible effect on the feed conversion ratio.

Regarding the mortality rate of broilers, non-challenged broilers given *Lawsonia inermis* powder did not exhibit any mortalities. These results align with those of Kiavandani et al. [35] who found that addition of *Lawsonia inermis* in the diets of broilers diet did not affect the mortality rate. Antifungal, antibacterial, virucidal, antiparasitic, and anti-inflammatory properties of *Lawsonia inermis* may be responsible for this outcome [44, 45]. It was reported that *Acacia nilotica* exerts hepato-protective effects by providing maximum protection against Carbon tetrachloride (CCl4)-induced liver injury [46, 47]. Additionally, the polyphenol extracted from the bark of *Acacia* species lowered fat accumulation in the liver [48]. Consequently, the above points clearly showed that *Acacia nilotica* was known to has hepato-protective property in birds. Moreover, it was demonstrated that supplementation of polyherbal formulation containing *Acacia nilotica* with choline chloride (CCL 60%) at finisher stage facilitates the fat mobilization from the liver in the broiler chickens and in turn prevent fatty liver condition and decrease mortality rates [49]. Results of mortality rates observed in challenged broilers fed on *Lawsonia inermis* agree with the findings obtained by Anna et al. [50] who investigated the anti-coccidial qualities of a 90% ethanolic extract of *Lawsonia inermis* leaves against caecal coccidiosis in broilers and in comparison with salinomycin, the 300-ppm dose of *Lawsonia inermis* leaf extract as a feed supplement demonstrated strong anti-coccidial activity and significantly reduced lesions and mortality rates. Regarding to Acacia nilotica which reduced the mortality rate in challenged broilers, Zahid et al. [18] indicated that *Acacia nilotica* bark extract contains several active chemicals that protect broilers under pathological conditions as done by antibiotics. As previously mentioned, *Acacia nilotica* bark extract contains terpenoids, alkaloids, glycosides, tannins, and saponins that have antibacterial properties against *E. coli* and other pathogenic bacteria. Additionally, saponins aid in the absorption of nutrients and medications, whereas tannins help to prevent the development of microorganisms [51]. All these points suggested that the extract of *Acacia nilotica* could improve the livability in boilers as supported by antibiotics. In addition, Abudabos et al. [52] showed that extract and phytogenic feed additives obtained from different herbs and spices could support the health in boilers under pathologically challenged or unchallenged conditions [53, 54].

### Biochemical metrics

*Lawsonia inermis* contains bioactive naphthoquinone, which was studied by [55]. We looked at the hepato-protective effects by evaluating the blood levels of ALT and AST in both challenged and non-challenged groups with coccidia, which considerably reduced as findings of Batiha et al. [56]. According to Abdulfatai and Ayotunde [57] as a result of *LIP*’s nephron-protective effects, we found that blood urea and creatinine did not significantly rise in the *LIP* treated groups that were either challenged with coccidia or not. Phytochemicals component of flavonoids and polyphenolics, which are potent antioxidants and may greatly contribute to the nephron-protective effects that safeguard the kidneys from damage caused by challenged or non-challenged groups with coccidia. Furthermore, a prior investigation [56], in addition to its anti-parasitic and hypoglycemic properties, *Lawsonia inermis* has anti-inflammatory, analgesic, and antioxidant properties that help avoid hepatotoxicity, gastro-protective and anticancer effects. *Lawsonia inermis’s* ability to prevent hepatotoxicity in mice revealed that it significantly raised glutathione levels that had been decreased. *Acacia nilotica* treatment was superior to *Lawsonia inermis* in lowering increased serum urea and creatinine levels. These results may be due to renal biomarkers decrease when *ANAE* is consumed, suggesting improved glomerular function [58]. Also, triglycerides, cholesterol, and glucose levels in the challenged group with or without coccidia are normalized by *LIP* due to phytochemical components of *LIP* as expressed by Batiha et al. [56]. Serum protein is the total amount of protein in the blood in which albumin is the most abundant. Albumin helps keep the blood from leaking out of blood vessels as well as to carry some medicines and other substances through the blood that is important for tissue growth and healing. Low total protein levels in all challenged groups in comparison with non-challenged groups can suggest a liver disorder, a kidney disorder, or a disorder in which protein is not digested or absorbed properly. By addition of *LIP* and *ANAE* alone or in combination in the diets of challenged broilers increased serum total protein and its fractions (albumin and globulin). This results are agree with the findings of Tijani et al. [59] Who found that the varying inclusion level of *Lawsonia inermis* leaf meal in the feed of the birds have significant (*P* > 0.05) effect on the serum biochemistry parameters.

Blood biochemistry study revealed that *ANAE* lowered blood glucose levels, increased albumin levels, and preserved a normal blood lipid profile in broilers. These results are consistent with the findings of Mukundi et al. [60]. The lipid profile, which includes triglycerides, total cholesterol, plus liver function markers(ALT&AST), significantly decreased in compared with G2 (positive control group), as indicated by Daud et al. [36]. We found that *ANAE* reduced blood glucose and lipid levels in both challenged and non-challenged groups with coccidia. Epicatechin, the main component of *Acacia nilotica*, inhibits pancreatic lipase activity in the gastrointestinal lumen, which reduces lipid absorption. In addition to regulating lipid absorption, epicatechin also controls transcription factors expressed during the synthesis of TG and cholesterol, including sterol regulatory element-binding protein and peroxisome proliferators-activated receptor gamma. Additionally, by preventing inflammation in white adipose tissue and, consequently, lipolysis as shown by Khalaf et al. [61], epicatechin was able to lower the amount of free fatty acids (FFA) in circulation. It was demonstrated that the folk remedy *ANAE*, which has strong antioxidant properties and has been demonstrated to exhibit a range of biological activities, including hepato-protective and neuroprotective qualities as well as lowering the level of biochemical parameters [48] associated with both coccidial-challenged groups and non-challenged groups, restored the normal architecture of hepatocytes when supplemented with *Acacia* species [62].

### Oxidative stress parameters

Lipid peroxidation and cytotoxic alterations are brought on by oxidative stress which caused by coccidia. These alterations compromises the integrity of the gastrointestinal tract and destroys the intestines [63]. *Lawsonia inermis* has antifungal, antibacterial, virucidal, anti-parasitic, and anti-inflammatory activities, which could account for this result [44, 45]. It has been demonstrated that *Lawsonia inermis* has potent antioxidant action reduces or eliminates the generation of free radicals via lowering MDA. It is thought to be a safe herbal remedy with very few and minor side effects. The bioactive constituents found in the aqueous *Acacia nilotica* extracts, such as flavonoids, polysaccharides, and organic acids, may be principally responsible for their pharmacological effects. In addition to having reduced levels of free radicals and MDA, the chickens treated with *Acacia extract* whether challenged or not had increased levels of antioxidants including SOD and GPX, which are used to scavenge and eliminate free radicals. These results were corroborated by [58].

MDA is a sign of radical-induced oxidative stress, and SOD aids in the elimination of reactive oxygen species. Meanwhile, the balance between ROS and the host’s ability to neutralize them was upset during the Eimeria infection due to host-immune responses, indicating higher oxidative stress during coccidiosis. GSH and GSSG are capable of preventing damage to critical cellular components caused by ROS [64]. Herbs with tannin-containing anti-coccidial properties can shield intestinal health, diminish the adverse effects caused by Eimeria-infected poultry, and promote the growth of broiler chickens. They can also increase the antioxidant capacity (SOD and GPx) in the serum and tissues [65] and resulting in drop in oxidative stress-related indices [66].

### Cytokines biomarkers

According to Kumar et al. [67] this incidence demonstrates the antioxidant defense of *Lawsonia inermis* against toxicants that cause organ damage. The serum levels of inflammatory markers such TNF-α and IL-4 were reduced in the broiler groups, as reported by Rakhshandeh et al. [68] whom suggested that *LIP*’s anti-inflammatory and antioxidant properties may have neuroprotective effects. Furthermore *Lawsonia inermis* has demonstrated anti-inflammatory and antioxidant qualities, which have been linked to its ameliorative effect by inhibiting inflammatory markers such as IL-4 [69], and TNF-α [70]. Consequently, these findings account for the rise in anti-inflammatory cytokines such as IL-10.

The tannins and quercetin components of *ANAE* are responsible for *Acacia nilotica’s* ability to inhibit inflammation in chicken groups that were challenged and those that were not. The anti-inflammatory cytokine IL-10 was increased in chicken serum by *ANAE*, while the inflammatory factors TNF-α and IL-4 were inhibited in their release as mentioned by Waseem et al. [71]. Based on our results, it is evident that *ANAE* should be taken into consideration as a viable substitute for antibiotic growth promoters in broiler diets. This is consistent with the conclusions of according to Abdel-Wareth et al. [72]. Accordingly, *LIP* and *ANAE* both work well as naturally occurring herbal boosters of chicken development in our experiment. The production of pro-inflammatory cytokines like TNF-α, increased by stimulating macrophages, which are the primary source of these cytokines throughout intrinsic infection phases. But IL-10 lessens and reducing the production of pro-inflammatory cytokines [63]. Medicinal plants and herbs may be crucial in helping animals’ immune systems function better. When comparing the antioxidant activity of the *LIP* groups to the control, they were found to have significantly lessened the accumulation of oxidative species in broiler chicks when challenged with or without coccidian [56]. The oxidative stress response, which is manifested by a decline in antioxidant enzymes and an elevation in pro-inflammatory cytokines, is triggered by *Eimeriatenella* [73]. Major modulators of adaptive immune responses include the cytokines as inteleukin-4 (IL-4). When hens are infected with various strains of *Eimeria*, their protein expression of IL-4 will increase. IL-4 promotes B cells to convert IgE and create major histocompatibility complex-II (MHC-II) molecules, as well as to multiply and develop into plasma cells as mentioned by Zheng et al. [74].

Herbs that possess anti-coccidial properties due to their tannin content can lower inflammatory cytokines like TNF-α in peripheral serum and boost the body’s defenses against infection (IL-10 and IgA) [65]. Tannin-containing herbs have been shown to activate the Nrf2/Keap1/HO-1 signaling pathway in chicks, leading to a reduction in indices associated with oxidative stress and an enhancement in the activity of antioxidant enzymes such as SOD, and GPX. Additionally, the observed down regulation of inflammatory cytokines further substantiates these findings [66].

### Oocyst shedding (total oocyst count)

Recent studies have shown that the biological activities of the natural ingredients in herbal extracts can effectively treat avian coccidiosis [75]. Avian anti-coccidial phytochemicals improved T cell-mediated immunity by preventing oocyst sporulation and sporozoite invasion [76]. Numerous secondary metabolites are present in plants with pharmacological properties that can be used to manage and treat a wide range of disorders. *Lawsonia inermis* powder decreased the oocyst number in our experiment according to Anna et al. [50], broiler chickens were given *Lawsonia inermis* at a level of 300 ppm as an anti-coccidial drug, which reduced the number of lesions and bird mortality. The decreased quantity of coccidian oocysts has been linked to *Acacia nilotica* aqueous extract (*ANAE*), which is rich in phenolic compounds and alkaloids as confirmed by [77]. Our findings demonstrated the potent inhibitory effects of *LIP* and *ANA*E, particularly when used together, on oocyst sporulation. Our findings were corroborated by Balta et al. [78], who discovered that natural antibacterial mixtures can hinder parasites’ ability to proliferate. Many herbal plants have been shown to raise the fraction of degenerated oocysts and lower the ratio of sporulated oocysts as confirmed by Yong et al. [79]. Consequently, medicinal plants serve as crucial for the development and progress of current research on the biological activities of mix of *LIP* and *ANAE* prevent coccidia proliferation in the chicken intestine and subsequently their drop.

### Intestinal histopathology and lesion scores

Histologically, the birds in the infected control group without improvements showed damage to their intestinal enterocytes, which has a major impact on their ability to absorb and use nutrition. Moreover, the mucosa’s intact intestinal layouts in groups that received both *LIP* and *ANAE* in Group 5B showed that oocysts die as a result of interactions between phenolic chemicals present in herbal extracts and the cytoplasmic membranes of coccidia. In addition, these substances can promote the repair of intestinal lipid peroxidation, reduce intestinal permeability caused by Eimeria, and restore damaged epithelial cells as confirmed by Nahed et al. [1]. In order to maximize growth performance and, thus, increase the efficiency of chicken production, gut health is crucial [80]. According to Lien et al. [81], the invasion of Eimeria species was found to cause an imbalance in the gut that had a negative impact on the internal mucosa. However, the phytogenic additives improved the intestinal microbiotia of chickens challenged with coccidia by decreasing the severity of lesion scores in the small intestine and cecum. The unique effect of *Lawsania inermis* powder and *Acacia nilotica* extract in the management of coccidiosis is well supported by this investigation.

## Conclusion

Dietary supplementation of *Lawsonia inermis* powder at a dosage of 40 mg/kg feed and *Acacia nilotica* extract at a dosage of 5 g/kg feed, either separately or in combination suggests that the combination of *LIP* and *ANAE* had more beneficial effects on growth performance, biochemical parameters, antioxidant status, cellular response, and intestinal morphology of broiler chickens when exposed to Eimeria spp. parasite, followed by *ANAE*, then *LIP* alone. Thus, both *LIP* and *ANAE* could be used as a potential natural anti-coccidial in broilers nutrition.

## Data Availability

Data is provided within the manuscript.

## Abbreviations

ADG: Average daily gain
AGPs: Antibiotic growth promoters
Alb: Albumin
ALT: Alanine transaminase
ANAE: Acaccia nilotica aqueous extract
AST: Aspartic transaminase
BUN: Blood urea nitrogen
BW: Body weight
BWG: Body weight gain
CF: Crude fiber
CRE: Creatinine
CP: Crude protein
DM: Dry matter
EE: Ether extract
EBI: European Broiler Index
EPEF: European Production Efficiency Factor
FCR: Feed conversion ratio
GPX: Glutathione peroxidase enzyme
GSSG: Oxidized glutathione
FI: Feed intake
H & E: Hemotoxylin stain
IL-4: Interleukin-4
IL-10: Interleukin-10
LIP: Lawsonia inermis powder
MHC-II: Major histocompatibility complex-II
MDA: Maloenaldehyde
NFE: Nitrogen free extract
OPG: Oocyst per gram
PI: Performance index
ROS: Reactive oxygen species
SOD: Super Oxide Dismutase
TG: Triglycerides
TNF-α: Alpha tumor necrosis factor
TP: Total protein
TWG: Total weight gain
WG: Weight gain
W/V: Weight by volume
